# Outpatient total hip and knee arthroplasty – Patient expectations versus experience

**DOI:** 10.1016/j.jor.2024.01.014

**Published:** 2024-02-01

**Authors:** Justin D. Bric, Emilie N. Miley, Hari K. Parvataneni, Luis Pulido, Hernan A. Prieto, Chancellor F. Gray, Justin T. Deen

**Affiliations:** a The Southeast Permanente Medical Group, Atlanta, GA, USA; b Department of Orthopaedic Surgery, College of Medicine, University of Florida, Gainesville, FL, USA; c Florida Orthopedic Institute, Gainesville, FL, USA

**Keywords:** Outpatient total hip arthroplasty, Outpatient total knee arthroplasty, Outpatient hip replacement, Outpatient knee replacement, Patient experience

## Abstract

**Aims & objectives:**

With modern advancements in surgical techniques and rapid recovery protocols, incidence of outpatient total joint arthroplasty (TJA) is increasing. Previous literature has historically focused on cost, safety, and clinical outcomes, with few studies investigating patient expectations and experiences. The aim of this study was to survey preoperative patient expectations related to outpatient TJA surgery compared with perioperative perceptions and experience.

**Materials & methods:**

Prospective study of patients undergoing outpatient total hip or knee arthroplasty at a single Tertiary Academic center. Preoperative and postoperative surveys were administered during routine clinic visits.

**Results:**

One hundred and six patients completed preoperative surveys; 79 completed postoperative surveys and were included in the final data analysis. Fifty (63.3 %) patients reported being aware of outpatient TJA prior to undergoing the procedure. There was no difference between preoperative anticipated pain control and postoperative perceived pain control (6.64 vs. 6.88, p = 0.77). Most postoperative patients (N = 56, 70.9 %) rated outpatient surgery as “much better” or “better” than expected. Most postoperative patients (N = 68, 86 %) would opt to have outpatient surgery again. Fifty-two (65.8 %) of postoperative patients believed outpatient surgery sped up their postoperative rehabilitation.

**Conclusion:**

For most patients, the outpatient surgical experience met or exceeded expectations. Nearly 90 % of patients would prefer to have outpatient surgery in the future, further supporting the continued migration of elective arthroplasty away from inpatient sites of care.

## 1 Introduction

With significant projected volume increases for total knee arthroplasty (TKA) and total hip arthroplasty (THA) over the next decade, outpatient total joint arthroplasty (TJA) has emerged as an option to meet expected growth while preserving value.^1^^,^^2^ Outpatient protocols evolved naturally with the wide implementation of Enhanced Recovery After Surgery (ERAS) protocols. This is beneficial to the patient, as it mitigates costly, high-risk inpatient interactions and facilitates an early return to home. These important advantages were uniquely highlighted during the events of the COVID-19 Pandemic.^3^ Historically, patients progress through standardized, streamlined perioperative pathways designed to decrease complications, improve outcomes, and increase patient satisfaction.^2^^,^^4^^,^^5^ In addition, outpatient surgery can also be beneficial to the surgeon and the health care system as a means of reducing cost and resources as compared to traditional inpatient procedures.^6^, ^7^, ^8^, ^9^ Current clinical outcome data shows that outpatient TJA is safe, with complication rates similar to inpatient TJA.^1^^,^^2^^,^^4^^,^^10^^,^^11^ Additionally, patient-reported outcome measures have also been reported to be similar between the sites of care.^2^^,^^11^

Successful implementation of an outpatient TJA program has been described to have four primary aims: quality, safety, affordability, and patient experience.^5^ While there has been a strong scientific and policy focus on the surgeon, hospital, and outcomes of outpatient TJA, there has been limited reporting on the patients' experience. Current series available are limited to either preoperative or postoperative samples, or retrospective reviews of patients who previously received inpatient TJA.^7^^,^^8^^,^^11^ To our knowledge, minimal studies have prospectively followed patients through an outpatient TJA pathway and compared the patients’ preoperative expectations with their actual surgical experience. As such, the aim of this study was to survey preoperative patient expectations related to outpatient TJA surgery compared to actual perioperative perceptions and experience. We hypothesize patients perioperative experience will be meet or exceed their preoperative expectations.

## 2 Methods

### 2.1 Study design

A prospective, cohort study was performed at our institution.

### 2.2 Setting

**Fig. 1:**
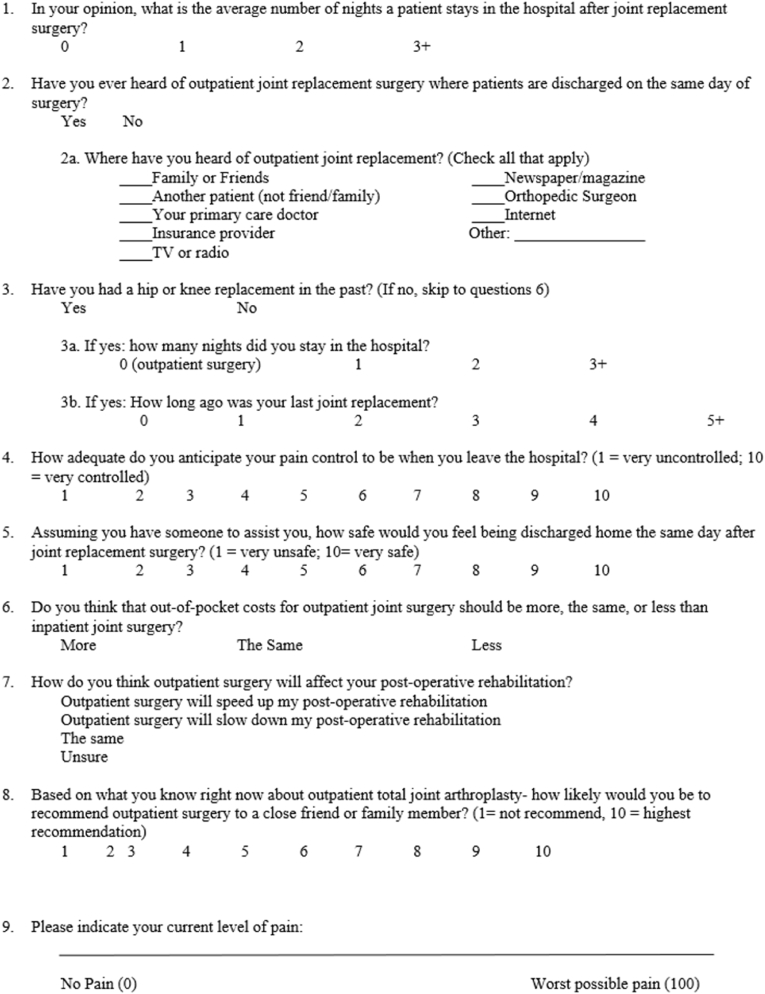
Preoperative survey.

**Fig. 2:**
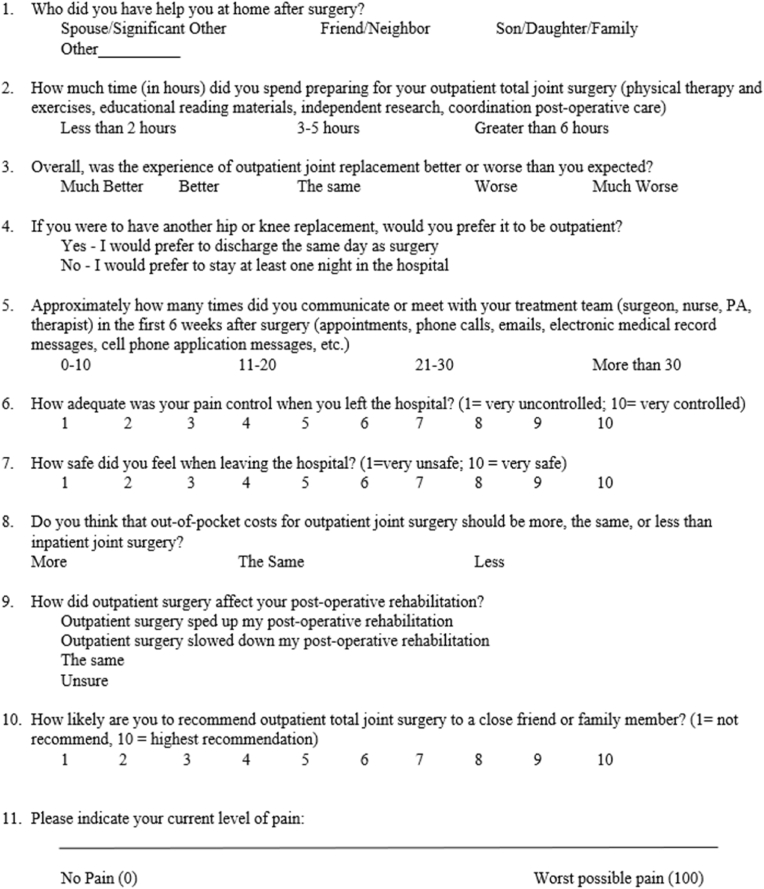
Postoperative survey.

Patient recruitment occurred in a clinic setting at an academic orthopaedic department. All consecutive patients scheduled for THA and TKA over the study time frame were screened. Patient enrollment occurred at the time of the patient's decision for surgery. Consent and preoperative survey (Fig. 1) completion was administered at the conclusion of the visit, but prior to scheduling, disposition discussions, preoperative education, and/or counseling (i.e., to avoid bias of the surgical education program and counseling on perception). In addition, a postoperative survey (Fig. 2) was administered at the first scheduled visit postoperatively.

### 2.3 Participants

Patients were prospectively screened for this study if they were undergoing elective unilateral primary TKA or THA and met institutional criteria for outpatient surgery.^9^ Exclusion criteria included: 1) patients who received a revision or conversion arthroplasty, 2) patients receiving concurrent bilateral surgery, 3) patients who were undergoing surgery due to infection, 4) and patients less than 18 years of age. Patients then underwent surgery through our outpatient surgical pathway (same day discharge) as scheduled within our academic tertiary care hospital. In this setting, procedures are performed at the same site regardless of inpatient or outpatient status, but patients who met outpatient criteria are discharged home from the recovery room. All patients received a scheduled standardized multimodal perioperative pain regimen (i.e., acetaminophen, NSAID, gabapentinoid therapy) in addition to one of four of our institutional stratified opioid prescription pathways.^12^ Prescribing practices were independent of discharge disposition. Outpatient discharge criteria included hemodynamic stability, spontaneous urination, tolerating diet, pain control on oral medication, and physical therapy criteria of safe ambulation.

### 2.4 Variables

Demographic data (i.e., sex, age, procedure type, body mass index [BMI], Risk Assessment and Prediction Tool [RAPT], and insurance type) were collected for this study. In addition, surveys administered were collected at the preoperative and preoperative visit. See Fig. 1, Fig. 2 for the corresponding survey.

### 2.5 Data sources/management

Survey questions were developed around common areas of apprehension, including pain control, safety, rehabilitation, and cost. Survey development and face validity occurred with collaboration between a group of physicians (i.e., five adult arthroplasty physicians with approximately 45 years of combined experience), one adult arthroplasty fellow, and a research faculty member (i.e., with approximately 33 years of research experience). After consensus, items were then converted to an online survey in REDCap (Research Electronic Data Capture; Vanderbilt University, Nashville, TN) prior to data collection. All surveys were administered through REDCap version 11.0.3 via tablet computer with the assistance of a single clinical research coordinator. Surveys were scored using a combination of Likert scale responses (i.e., ranked from 1 to 10) continuous scale (i.e., 0–100), and categorical responses (e.g., less, the same, more). Likert and continuous responses were used to compare preoperative and postoperative survey responses. There were a total of 9 questions in the preoperative survey (Fig. 1), and 11 total questions in the postoperative survey (Fig. 2).

### 2.6 Study size

A convenience sample was used for this study.

### 2.7 Statistical method

Continuous and Likert survey responses were reported as means and standard deviations (SD). Categorical survey responses were reported as total number of responses (N) and percent of overall responses. A Wilcoxon Signed-Rank test was used to compare preoperative and postoperative survey responses for the data that were ranked on a scale of one to ten (e.g., 1 = very unsafe, 10 = very safe). In addition, a Kruskal-Wallis was conducted to compare if any differences existed in survey responses using the calculated difference between preoperative and postoperative survey responses between groups (i.e., TKA and THA groups). For data that included categorical responses (e.g., more, the same, less) and were asked at two time points (i.e., preoperatively and postoperatively), a McNemar's test was conducted to determine if there was a difference between the two time points. Chi-square tests (χ^2^) were conducted on the preoperative responses between groups (i.e., TKA and THA) and postoperative responses between groups. Lastly, a repeated-measures analysis of variance (RM-ANOVA) was conducted to determine if any differences existed between patient's pain level across two time points and between groups. Patients who were admitted postoperatively or lost to follow-up were excluded from this study.

## 3 Results

### 3.1 Participants

A total of 238 patients were screened for inclusion of this study. Of the patients screened, a total of 113 patients agreed to participate and were enrolled. Thirty-four patients were excluded for not completing the postoperative survey due to the following reasons: lost to follow-up (N = 10), never scheduled surgery (N = 6), cancelled surgery (N = 6), rescheduled surgery outside the study timeframe (N = 6) or were admitted to the hospital following surgery (N = 6). The final sample consisted of a total of 79 patients who completed both surveys (i.e., preoperatively, postoperatively) and were included in the final analysis.

### 3.2 Descriptive data

Of the patients included in the final data set, 51 (64.6 %) patients underwent a TKA, and 28 (35.4 %) patients underwent a THA. In addition, 46 (58.2 %) patients were females and 33 (41.8 %) were males. The mean Risk Assessment Prediction Tool (RAPT) score, a score used to predict patient's disposition after the hospital, was 9.23 ± 1.71 ^3^^,^^13^. Additional demographic data is listed in Table 1. The average time between the preoperative survey and surgery was 9.59 ± 5.28 (range = 1.00–21.86) weeks, between surgery and administration of the postoperative survey was 6.33 ± 2.84 (range = 1.86–16.29) weeks, and preoperative survey to postoperative survey was 15.92 ± 5.55 (range = 4.00–27.00) weeks.

**Table 1:** Demographic information.

	TKA (N = 51)	THA (N = 28)	Overall (N = 79)
**Sex** N (%)			
Male	20 (60.6)	13 (39.4)	33 (41.8)
Female	31 (67.4)	15 (32.6)	46 (49.5)
**Age** (years ± SD)	66.24 ± 7.90	67.27 ± 12.77	66.60 ± 9.83
**Procedure Type** N (%)	51 (64.6)	28 (35.4)	79 (100)
**BMI** (mean ± SD)	31.91 ± 6.05	30.42 ± 7.08	31.38 ± 6.42
**RAPT** (mean ± SD)	9.42 ± 1.50	8.89 ± 2.02	9.23 ± 1.71
**Insurance Type** N (%)			
Medicare	31 (60.8)	16 (57.1)	47 (59.5)
Medicaid	9 (17.6)	3 (10.7)	12 (15.2)
Commercial	10 (19.6)	2 (7.1)	17 (21.5)
Self-Pay	0 (0.0)	2 (7.1)	2 (2.5)
Other	1 (2.0)	0 (0.0)	1 (1.3)

### 3.3 Main results

Prior to surgery, 50 (63.3 %) patients reported that they were aware of outpatient TJA (Table 2). The most common sources for exposure were family/friends (32.9 %) and Orthopaedic Doctor (27.8 %). Nineteen (16.8 %) patients had a prior TJA, none of which were prior outpatient surgery. Preoperatively, 49 (62.0 %) of patients responded that outpatient TJA should have less out of pocket costs than inpatient, while 25 (31.6 %) patients responded it should be the same. After surgery, responses were N = 32 (40.5 %) and N = 43 (54.4 %), respectively.

**Table 2:** Preoperative descriptive survey responses.

Question	TKA N (%)	THA N (%)	P-value
In your opinion, what is the average number of nights a patient stays in the hospital? (N = 74)			
0	0 (0.0)	0 (0.0)	**0.03**
1	38 (79.2)	13 (50.0)	
2	7 (14.6)	7 (26.9)	
3	3 (6.3)	6 (23.1)	
Have you ever heard of outpatient joint replacement surgery where patients are discharged on the same day of surgery? (N = 74)			
Yes	32 (66.7)	18 (69.2)	0.82
No	16 (33.3)	8 (30.8)	
Where have you heard of outpatient joint surgery? (N = 79)			
Family/Friends	18 (35.3)	8 (28.6)	
Another Patient (Not family/friend)	7 (13.7)	2 (7.1)	
Primary Care Physician	4 (7.8)	5 (17.9)	
Insurance Provider	0 (0.0)	0 (0.0)	*
TV or Radio	2 (3.9)	2 (7.1)	
Newspaper or Magazine	1 (2.0)	0 (0.0)	
Orthopaedic Surgeon	17 (27.5)	8 (28.6)	
Internet	1 (2.0)	1 (3.6)	
Other	1 (2.0)	1 (3.6)	
Have you had a hip or knee replacement in the past? (N = 75)			
Yes	11 (22.4)	8 (30.8)	0.43
No	38 (77.6)	18 (69.2)	

There was a significant difference identified between TKA and THA patients (χ^2^ = 7.37, p = 0.03) regarding question one (i.e., “In your opinion, what is the average number of nights a patient stays in the hospital after joint replacement surgery”) on the preoperative survey (Table 2), with the THA group reporting a longer perceived stay than the TKA group. There were no other significant differences in the preoperative survey responses between THA and TKA groups (p > 0.05). Additionally, there were no differences identified between the TKA and THA groups for the postoperative survey responses (Table 3).

**Table 3:** Postoperative descriptive survey responses.

Question	TKA N (%)	THA N (%)	P-value
Who did you have help you at home after surgery? (N = 79)			
Spouse/Significant Other	32 (62.7)	19 (67.9)	0.08
Friend/Neighbor	1 (2.0)	4 (14.3)	
Son/Daughter/Family	16 (31.4)	5 (17.9)	
Other	2 (3.9)	0 (0.0)	
How much time in hours did you spend preparing for your outpatient total joint surgery? (N = 79)			
Less than 2 h	20 (39.2)	10 (35.7)	0.82
3–5 h	18 (35.3)	9 (32.1)	
Greater than 6 h	13 (25.5)	9 (32.1)	
Overall, was the experience of outpatient joint replacement better or worse than you expected? (N = 79)			
Much Better	15 (29.4)	14 (50.0)	
Better	18 (35.3)	9 (32.1)	
The same	13 (25.5)	3 (10.7)	0.24
Worse	5 (9.8)	2 (7.1)	
Much Worse	0 (0.0)	0 (0.0)	
If you were to have another hip or knee replacement, would you prefer it to be outpatient? (N = 68)			
Yes	44 (100.0)	24 (100.0)	*
No	0 (0.0)	0 (0.0)	
Approximately how many times did you communicate or meet with your treatment team in the first 6 weeks after surgery? (N = 79)			
0-10	31 (60.8)	21 (75.0)	
11-20	15 (29.4)	5 (17.9)	0.44
21-30	5 (9.8)	2 (7.1)	
More than 30	0 (0.0)	0 (0.0)	

There was no difference between preoperative anticipated pain control and postoperative perceived pain control upon discharge (7.14 vs. 7.18, p = 0.77; Table 4). Also, there was no significant difference between anticipated safety at discharge preoperative and postoperatively (8.01 vs. 8.49, p = 0.27; Table 4). There was a significant difference in pain rating from preoperatively to postoperative between the TKA and THA groups, consistent with existing evidence.^17^ The TKA group improved from 59.98 ± 18.20 to 30.60 ± 22.98, while the THA group improved from 54.74 ± 25.32 to 10.04 ± 13.30 (p = 0.04, Table 4).

**Table 4:** Preoperative and postoperative survey responses.

Question	TKA Mean ± SD	THA Mean ± SD	P-value
How adequate do you anticipate/was your pain control when you leave the hospital?			
Pre	6.06 ± 2.82	7.73 ± 2.46	0.77^a^
Post	6.51 ± 3.09	7.29 ± 3.35	
How safe would you feel being discharged home the same day/did you feel when leaving the hospital?			
Pre	7.86 ± 3.29	8.31 ± 2.75	0.27^a^
Post	8.76 ± 2.36	8.00 ± 3.43	
How likely are you to recommend outpatient surgery to a close friend or family member?			
Pre	8.17 ± 2.45	8.46 ± 2.06	0.14^a^
Post	8.56 ± 2.78	8.88 ± 2.36	
Please indicate your current level of pain.			
Pre	59.98 ± 18.20	54.74 ± 25.32	**0.04**^b^
Post	30.60 ± 22.98	10.04 ± 13.30	
Do you think out of pocket costs for outpatient joint surgery should be more, the same, or less than inpatient joint surgery? (N = 75)	N (%)	N (%)	
Pre			
More	1 (2.0)	0 (0.0)	
The Same	15 (30.6)	10 (38.5)	
Less	33 (67.3)	16 (61.5)	
Post			0.50^c^
More	3 (5.9)	0 (0.0)	
The Same	25 (49.0)	18 (66.7)	
Less	23 (45.1)	9 (33.1)	
How did outpatient surgery affect your postoperative rehabilitation?			
Pre (N = 84)			
Outpatient surgery will speed up my rehabilitation	21 (42.9)	10 (38.5)	
Outpatient surgery will slow down my rehabilitation	1 (2.0)	0 (0.0)	
The Same	18 (36.7)	10 (38.5)	
Unsure	9 (18.4)	15 (20.0)	
Pre (N = 79)			**< 0.01**^c^
Outpatient surgery sped up my rehabilitation	35 (68.6)	17 (60.7)	
Outpatient surgery slowed down my rehabilitation	4 (7.2)	1 (3.6)	
The Same	5 (9.8)	5 (17.9)	
Unsure	7 (13.7)	5 (17.9)	

Bolded = Statistically significant finding.

a Wilcoxon Signed-Rank test statistic.

b Repeated-Measures ANOVA test-statistic.

c McNemar's test-statistic.

Additionally, 56 (70.8 %) of patients postoperatively rated outpatient surgery as “much better” or “better” than expected (Table 3). Also, 52 (65.8 %) of patients postoperatively believed outpatient surgery sped up their post operative rehabilitation, compared to 31 (36.9 %) that anticipated it would preoperatively. Lastly, 68 (86 %) of postoperative patients would opt to have outpatient surgery again (Table 3).

## 4 Discussion

### 4.1 Key results

Public awareness of outpatient TJA is growing, and understanding the perceptions that our patients have is important as surgeons continue to shift more of their practices to this setting. A series of preoperative surveys in 2017 showed that 54.5 % of patients scheduled to undergo TJA were aware outpatient surgery was an option ^5^. In our study, this was 66.7 %, which is unsurprising considering increased publicity regarding outpatient surgery. However, literature in this space has historically focused on cost, safety, and outcomes, with few studies investigating the patient experience ^14^. To our knowledge, this is the first study to prospectively follow patients through an outpatient TJA pathway, with standardized preoperative and postoperative survey responses allowing for comparison of patients’ preoperative expectations against their actual operative experience.

Our results confirm the findings found by Meneghini et al. that patients generally perceive outpatient TJA will lead to faster recovery ^5^. In their series of 108 preoperative patient surveys, 61 % perceived that an advantage of outpatient surgery is faster recovery. Similarly, 65 % of our postoperative respondents indicated that outpatient TJA sped up their recovery.

In 2019, Barrack et al. collected responses from a series of 347 patients 6–12 months following an inpatient TKA, evaluating their perceptions of undergoing the same procedure as outpatient surgery. In their findings, 72 % reported either “Definitely not” or “Probably not” when asked if they would have been able to go home the same day after surgery ^15^. This is similar to a recent public crowdsourcing survey demonstrating that 64.6 % of the general public still prefers to stay in the hospital following TJA, and 68 % believe the hospital is the safest place for TJA.^14^ Additionally, results from a series of preoperative surveys indicate only 34 % of patients would be “very comfortable” or “comfortable” with same day discharge from TJA ^5^.

In contrast, our study indicates that most patients (70.8 %) had a better or much better experience than they expected. In addition, nearly 90 % would prefer to have outpatient surgery in the future. We believe the difference in these results compared to previously published series is due to our sampling of patients both preoperatively and postoperatively, offering the ability to highlight their experience exceeded expectations. The authors feel that this can be a powerful tool in the preoperative education of patients who are hesitant to undergo outpatient TJA and provides a mechanism of engaging patients and strengthening mutual decision making.

### 4.2 Limitations & strength

This study has limitations warranting discussion. First, it was a convenience sample of patients entering our outpatient TJA pathway and therefore was not powered. The nature of collecting survey responses resulted in different time frames of postoperative results collection, which may introduce recall bias. In addition, almost 25 % of preoperative surveys were excluded due to failure to complete a postoperative survey or because of postoperative admission, likely contributing to some form of selection bias. Lastly, the surveys were tested with face validity which is identified to be the weakest form of validity testing.

Interestingly, 14 % of patients did not prefer outpatient TJA, and 34 % did not perceive improved rehabilitation with outpatient surgery. The authors believe this represents an avenue for future research to better understand sources of variability of experience with outpatient surgery. Such barriers could also provide opportunities for educational and clinical pathway changes to ensure a consistent patient experience.

## 5 Conclusion

In patients undergoing outpatient hip and knee arthroplasty, there were no differences between preoperative expectations and postoperative experiences related to pain and safety. Most patients perceive quicker rehabilitation with outpatient TJA. For most patients, outpatient TJA experience was better than expected. Nearly 90 % of patients would prefer to have outpatient surgery in the future.

## Ethics statement

All procedures were performed in compliance with relevant laws and institutional guidelines and have been approved by the University of Florida's Institutional Review Board on November 23, 2021 (IRB#202102372). This study was approved by the university as an Expedited study.

## Funding source

This research was funded in part by 10.13039/100013622OMeGA (Grant #55255) and the NIH National Center for Advancing Transitional Sciences (10.13039/100006108NCATS) grant IL1 TR000064.

## Consent statement

Authors confirm that informed consent was obtained for all patients included in this study.

## CRediT authorship contribution statement

**Justin D. Bric:** Dr. **Emilie N. Miley:** contributed to manuscript preparation, Dr. **Hari K. Parvataneni:** contributed to manuscript preparation, Dr. **Luis Pulido:** contributed to manuscript preparation, Dr. **Hernan A. Prieto:** attributed the following to the following: reviewing the study, Conceptualization, assisted in gaining access to data, reviewed methods and results, and contributed manuscript preparation, Dr. **Chancellor F. Gray:** contributed to manuscript preparation, Dr. **Justin T. Deen:** confirms the following contributions to this study: study, Conceptualization, design, interpretation of results, and manuscript preparation. In addition, Dr.
